# Molecular Identification, Phylogenetic Status, and Geographic Distribution of *Culicoides oxystoma* (Diptera: Ceratopogonidae) in Israel

**DOI:** 10.1371/journal.pone.0033610

**Published:** 2012-03-16

**Authors:** Neta Morag, Yonatan Saroya, Yehuda Braverman, Eyal Klement, Yuval Gottlieb

**Affiliations:** Koret School of Veterinary Medicine, Robert H. Smith Faculty of Agriculture, Food, and Environment, The Hebrew University of Jerusalem, Rehovot, Israel; New Mexico State University, United States of America

## Abstract

*Culicoides oxystoma* (Diptera: Ceratopogonidae) is an important vector species, reported mainly from Asia, with high potential to transmit viral diseases affecting livestock. In Japan, many arboviruses have been isolated from *C. oxystoma*, suggesting it as a key player in the epidemiology of several *Culicoides*-borne diseases. Over the years, *C. oxystoma* has also been reported in the Middle East region, including Israel. In this region, however, *C. oxystoma* cannot be easily distinguished morphologically from its sibling species included in the *Culicoides schultzei* complex. We therefore used genomic data for species identification and phylogeny resolution. Phylogenetic analyses based on internal transcribed spacer 1 (ITS-1) of ribosomal DNA and the mitochondrial gene encoding cytochrome oxidase subunit I (COI) showed that *C. oxystoma* from Israel is closely related to *C. oxystoma* from Japan. Using differential probing PCR, we showed that *C. oxystoma* is distributed all over the country, especially in Mediterranean climate regions. *Culicoides oxystoma* is less common or even absent in arid regions, while the other genetic cluster of *C. schultzei* complex was found only in the east of the country (mostly arid and semiarid regions). The molecular finding of *C. oxystoma* in wide geographical regions, together with its high proportion in the general *Culicoides* population and its vectoring potential, imply that it may be an important vector species in the Middle East.

## Introduction

*Culicoides* biting midges (Diptera: Ceratopogonidae) are among the smallest hematophagous flies. They occur throughout the world, except New Zealand and the Antarctic. They play an essential role in the epidemiology of several major veterinary diseases as vectors of arboviruses which affect ruminants and equines [1]. In livestock, diseases caused by bluetongue virus (BTV) and epizootic hemorrhagic disease virus (EHDV), among others, are of international significance, and have an important impact on the economy and animal welfare [2], [3]. Several *Culicoides* species have been associated with the transmission of BTV and EHDV, hence restricting these diseases to areas where specific competent vector species occur and to their seasonal activity [4]–[6]. Since the intimate relation between a given virus and a vector determines the latter's competence [7], the taxonomic status of *Culicoides* vector species is essential for defying their role in disease epidemiology. Taxonomic identification of *Culicoides* species is based on morphological parameters that include wing pattern, measurements and proportions of antennal and palpal segments, counts of their various sensilae and genitalia [8]. This routinely used assessment can be time-consuming, particularly among *Culicoides* complexes, and is not always reliable for differentiating closely related species [9], [10]. Few taxonomists are able to distinguish roughly between sibling species, thus classification may result in misidentification [11]. It is therefore most efficient to use molecular and morphological methods in parallel, since specimen mounting is usually not efficient for downstream molecular procedures and vice versa.

Common and dominant *Culicoides* species recorded in Israel, in the eastern Mediterranean region and in Africa are *Culicoides imicola* and midges belonging to *Culicoides schultzei* complex [1], [12]. *Culicoides imicola* sensu stricto, the most important proven vector of BTV [1], [13], is quite easily identified by its typical wing pattern. Some members of the *C. schultzei* complex are also considered as potential vectors of BTV and EHDV [14]. This complex consists of several species described in the past (*C. oxystoma*, *C. schultzei*, *C. subschultzei*, *C. kingi*, *C. rhizophorensis*, *C. enderleini*, *C. nevilli* and *C. neoschultzei*) [15], [16]. Although the taxonomic status of *C. schultzei* complex has been studied [13], [15]–[20], the literature reveals confusing and inconsistent reports. Callot et al. [18] first reported on samples identified as *C. schultzei* Enderlein found in Israel which, several years later, were named kingi-schultzei gp. in light of the great morphological variability found among individuals [17]. Later, Cornet [19] described the discrepancies in this group and recorded six African species in a preliminary note. These species were then named and described in detail [15]. At that time, the taxonomic status of *Culicoides* BTV vectors was discussed by Wirth and Dyce [20], who referred to *C. schultzei* as a complex of species which requires further study. In that report, the identity of *Culicoides oxystoma*, a common species in Asia closely related to *C. schultzei*, was also stated as unclear. In Israel, *C. oxystoma* was reported as a member of the *C. schultzei* complex [12], [21]. Furthermore, three different morphological types of *C. oxystoma* were recorded, one of which was similar to the identified *C. oxystoma* sensu Arnaud from Japan [15]. Boorman [16] noted that the exact identity of this species is in doubt and evaluated that most of the records of *C. scultzei* group from north of the Sahara refer to *C. oxystoma*.

The inconclusive taxon status of *C. schultzei* complex, together with the expertise required to morphologically differentiate the members of this group, raise the need for less subjective identification methods that are based on genomic data. Although genomic records are unavailable for *C. schultzei* complex, previous studies have successfully initiated reliable molecular methods for the identification and definition of phylogenetic status of other *Culicoides* species. These methods are based on several genomic data including internal transcribed spacer 1 (ITS-1) and 2 (ITS-2) regions of ribosomal DNA [22]–[29], as well as the mitochondrial gene cytochrome oxidase subunit I (COI) and subunit II (COII) [10], [30]–[33] that are performed using regular PCR, real-time PCR, and micro-array. Recently, identification method based on mass-spectrometry was developed [34].

In this work, we applied molecular methods to simplify the identification of the commonly found *C. schultzei* complex, showing that some midges morphologically classified as *C. schultzei* complex from Israel are genetically closely related to *C. oxystoma* from Japan. As this species has the potential to transmit important arboviruses [16], [35], we analyzed its phylogenetic status and its geographical distribution to evaluate its vectoring potential in the Middle East region.

## Results

### Morphological identification of *Culicoides* species

**Figure 1:**
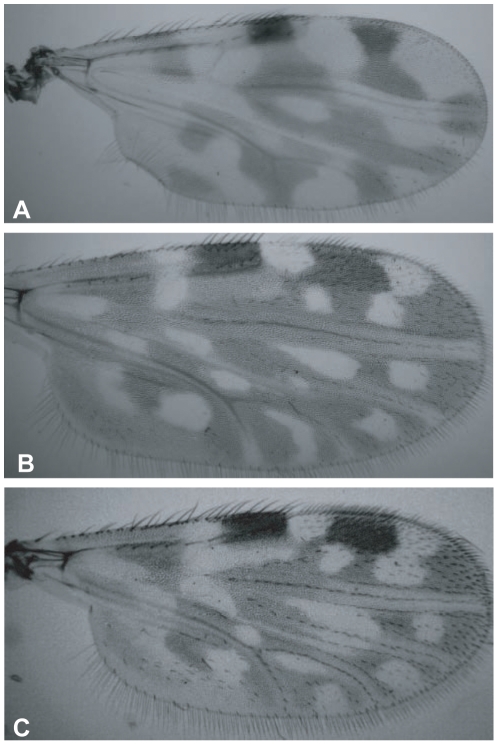
Photographs of wings of slide-mounted *Culicoides* specimens viewed under light microscope. Random samples of wing patterns of molecularly identified *C. imicola* (A), *C. oxystoma* (B) and another member of *schultzei* complex (C), demonstrating the difficulty in differentiating between species of the *schultzei* complex (B, C). Wing sizes from arculus to tip are 0.9 mm, 1.06 mm, and 0.93 mm, respectively.

A total of 217 specimens were classified based on wing patterns as members of the *C. schultzei* complex. These specimens could be easily morphologically differentiate from *C. imicola* found in the same collection batch. Although wing characterization was unique for each species, slight variations in wing pattern among individuals within a species was frequently observed (Figure 1). Due to the inconclusive taxon status of *C. schultzei* complex, together with the expertise required to morphologically differentiate its members, no attempt was made to distinguish between the species of this complex.

### Molecular identification and phylogenetic analyses of *Culicoides* species based on ITS-1, and COI gene

**Figure 2:**
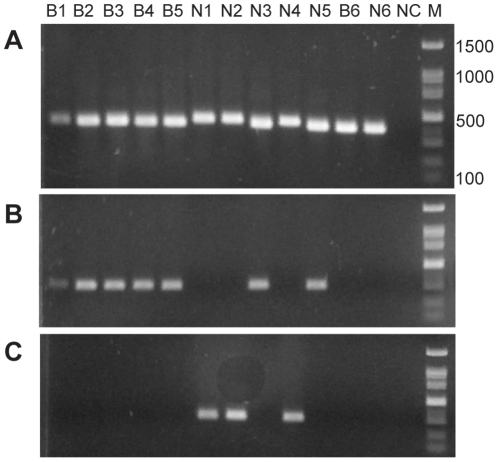
Diagnostic PCR of ITS-1 for *Culicoides* species. Agarose gels showing PCR samples of 10 individual midges morphologically identified as *C. schultzei* complex (B1–5, N1–5) and 2 individuals of *C. imicola* (B6, N6). Same DNA samples were used for phylogeny analysis (Figures 3,4). (A) Genus-specific PCR amplification (B) Species-specific PCR for identification of *C. oxystoma* (C) Species-specific PCR for identification of the other genetic cluster of *C. schultzei* complex. NC - negative control, containing no template; M - 100 bp DNA ladder.

Midges identified morphologically as *C. schultzei* complex (n = 10, two collection site: Newe-Ur, located in the Jordan Valley in a semiarid climate region, and Bet-Elazari, located in the center of the country in a Mediterranean climate region) were subjected to specific PCR using the ITS-1 primers. This PCR resulted in two different band sizes (Figure 2A) that were sequenced. The obtained sequences [GenBank: JN408469–JN408478] were closely related to the published sequence of *C. oxystoma* [GenBank: AB462279] [36]. The upper bands consisted of 420 to 423 bp and showed 87% similarity to *C. oxystoma*, whereas lower bands were 400 to 408 bp and showed 98% similarity to *C. oxystoma* (Figure 2A). According to sequence differences, species specific PCR for ITS-1 region was developed to differentiate between the first (Figure 2B) and the second (Figure 2C) genetic groups of the *C. schultzei* complex.

**Figure 3:**
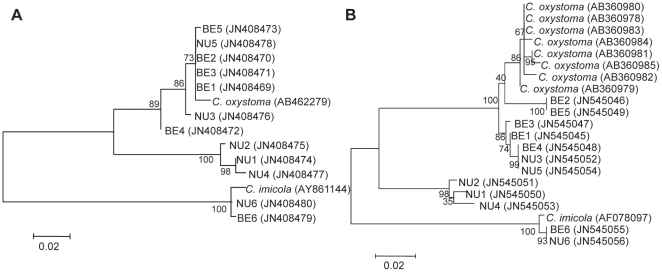
Maximum likelihood trees based on ITS-1 (A) and COI (B) nucleotide sequences obtained in this study. Trees show phylogenetic analyses of 10 individual midges morphologically identified as *C. schultzei* complex (BE1-5, NU1-5) and 2 *C. imicola* (BE6, NU6) collected from two sites together with most related published sequences.GenBank accession numbers are shown for each sequence. Midges were named by collection site (NU - Neve Ur, BE - Beit Elazari) with index numbers. Bootstrap values are shown on the branches.

**Figure 4:**
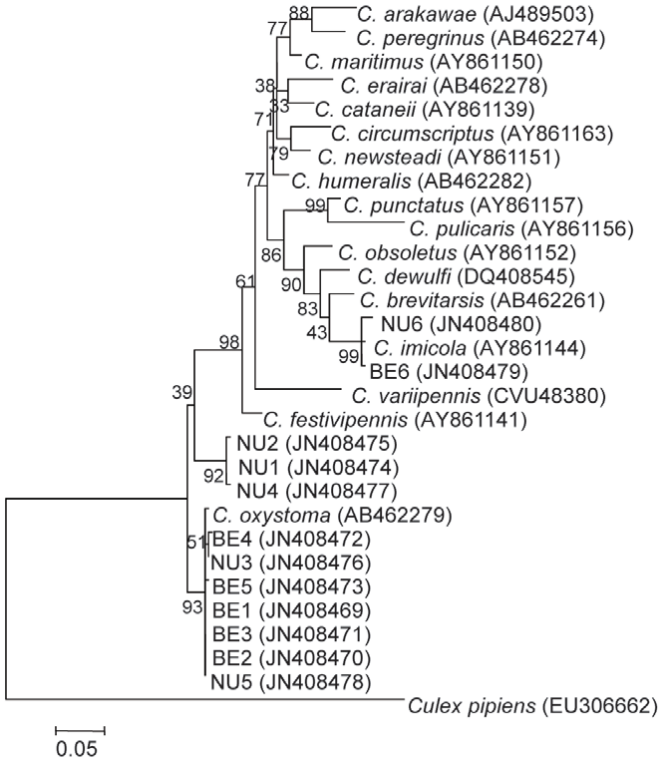
Phylogenetic analysis of different *Culicoides* species. Maximum likelihood phylogeny tree based on ITS-1 nucleotide sequences available in GenBank and 12 sequences obtained in this study (BE 1–6, NU 1–6). GenBank accession numbers are noted for each sequence. Bootstrap values are shown on the branches.

Phylogenetic trees generated with the two different methods: maximum likelihood (Figures 3A, 4), and neighbor joining (data not shown) revealed that the 10 individual midges identified morphologically as *C. schultzei* complex split significantly into two different genetic groups with an average of 83% similarity between the groups. One genetic group consisted of three midges collected in Neve-Ur (99.05% sequence homologies within the group) while the second genetic group consisted of seven midges collected from both locations (Neve-Ur and Beit- Elazari) (97.94% sequence homologies within the group) together with the available sequence of *C. oxystoma*.

Parallel PCR amplification of COI gene from the same specimens showed uniformity in band sizes (data not shown), yet their sequences [GenBank: JN545045–JN545054] revealed variation, showing between 90 and 98% similarity to the published sequence of *C. oxystoma* [GenBank: AB360979]. Consistent with the ITS-1 data, phylogenetic analysis based on the COI gene of the sequences obtained in this study and 8 *C. oxystoma* sequences from Japan [37], showed that midges (10 individuals) identified morphologically as *C. schultzei* complex in our study are significantly separated into two different genetic populations (Figure 3B) with an average of 91% similarity between the two groups. The first group which consisted of 3 individuals (99.3% sequence homologies within the group) showed 90% similarity to the published sequence of *C. oxystoma*, while the second group consisting of 7 individuals (99.01% sequence homologies within the group) showed 97–98% similarity to *C. oxystoma*.

Cloning and sequencing of the ITS-1 rDNA of *C. imicola* (two individual midges from the two different collection sites) revealed 392 bp sequences [GenBank: JN408479, JN408480] which were 99% identical to each other and to the published sequence of *C. imicola* from the island of Corsica, France [28] [GenBank: AY86114]. These two *C. imicola* sequences (Figures 3A, 4) were positioned together with another published sequence of *C. imicola* and branched in the same clade of other species belonging to subgenus *Avaritia* Fox: *C. brevitarsis*, *C. obsoletus* [28] (Figure 4). COI gene sequences obtained from the same *C. imicola* specimens [GenBank: JN545055, JN545056] showed 100% nucleotide sequence identity to each other and to several published *C. imicola* sequences from Israel and Europe [30], and these constituted a phylogenetically distinct population positioned in a separate clade from the members of the *C. schultzei* complex (Figure 3B).

### Species distribution within three climate regions

**Figure 5:**
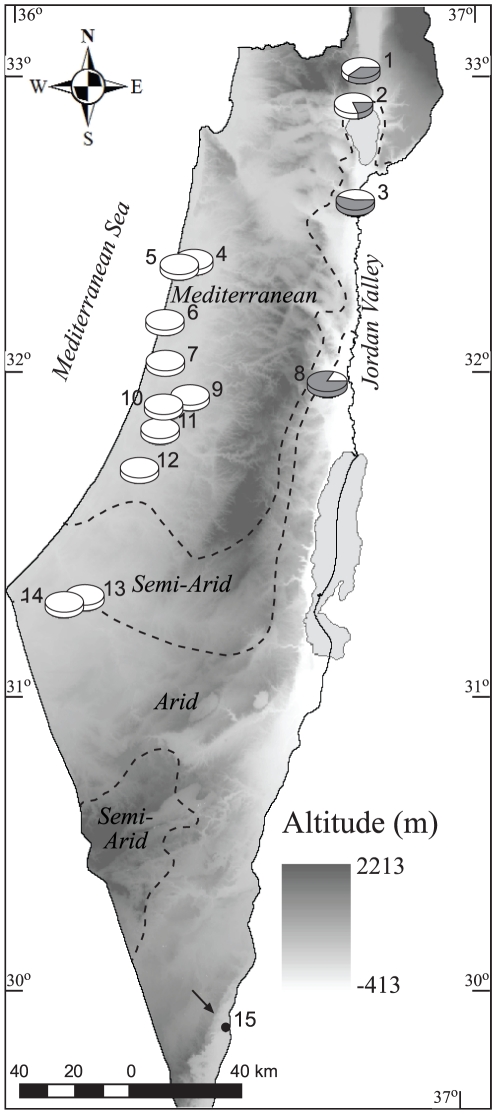
Topographic-climatic map of Israel showing the distribution of morphologically identified *Culicoides schultzei* complex (*n* = 217) sampled at 15 collection sites. Pie diagrams represent proportion of *C. oxystoma* (white) and *C. schultzei* gp. (dark gray) that were distinguished based on their ITS-1 sequence size. Arrow - collection site (Yotvata) where *C. schultzei* complex midges were absent. Numbers represent trap location sites (see table 1).

Based on ITS-1 band size which easily distinguished *C. oxystoma* from other *C. schulztei* complex species on an agarose gel, we determined the distribution of the two genetic groups in 15 settlements along three different climatic regions. *C. oxystoma* (n = 156) was shown to be widespread and prevalent in almost all geographical regions within the Mediterranean, semiarid, and arid climates, whereas a separate genetic cluster of the *C. schultzei* complex (n = 61) was found in the eastern part of the country (Figure 5). Both genetic clusters were less common or absent in the arid climate regions.

## Discussion

Despite much discussion of the taxonomic status of *C. oxystoma* as a member of the *C. schultzei* complex in Israel over the years, it remains unclear [12], [15]–[20]. It has been proposed to be the Asian analog of the Afrotropical species *C. subschultzei* [8]. Here we suggest that the molecularly identified *C. oxystoma* from Israel is closely related to *C. oxystoma* from Japan rather than to the other morphologically comparable and indistinguishable members of the *C. schultzei* complex found in Israel. These results are in agreement with the previously found morphological similarity of a *C. oxystoma* specimen from Israel to the identified *C. oxystoma* sensu Arnaud from Japan [15].

Variation between individual sequences in the same cluster and the phylogenetic analyses suggest that other genetic clusters within the *C. schultzei* complex are present that are not related to geographical location. Further analyses based on other genomic data may better resolve this complex. Our analyses can serve as a foundation to the future development of a simple identification method for other members of the *C. schultzei* complex.

Molecular identification of *C. oxystoma* is of great importance as this species is the most important potential vector of several arboviruses in Japan, with 85.9% of all viruses isolated from this species causing major livestock diseases, such as EHDV [35]. It would therefore be interesting to determine whether this important potential vector species plays a role in the epidemiology of *Culicoides*-borne diseases in Israel and the Middle East. In the past, members of the *C. schultzei* complex in Israel have been suspected as vectors for arboviruses [38]–[40]. Braverman et al. [21] evaluated the vectorial potential of three common species, including *C. oxystoma*, for BTV based on three criteria: the mean periods and survival rates between blood meals, and expectation of the species remaining infective throughout its life. Although *C. oxystoma* was present in the field for only a short period (ca. 2 weeks) during the BTV season, it showed the highest mean period between blood meals as well as the highest range of expectation to remain infective, suggesting that this species could serve as an important augmenting vector during that limited period. Although previous attempts to isolate arboviruses from pools of midges of the *C. schultzei* complex from Israel have been mainly unsuccessful [39], studies from other countries have demonstrated the isolation of arboviruses from this species complex [14], [35]. In addition, BTV was shown to multiply in an Israeli *C. schultzei* gp. midges following intrathoracic inoculation [38]. Therefore, further attempts to isolate viruses from recently field-collected *C. oxystoma* in Israel recent, as well as vector-competence experiments in the laboratory are required to determine the vectorial capacity of the *C. oxystoma* described in our study.

Other criteria which contribute to the vectorial capacity of *Culicoides* species are their prevalence, seasonality and geographical distribution. In this work, the molecularly identified *C. oxystoma* was dominantly found in almost all collection sites distributed in different geographical and climatic regions in Israel, suggesting this species' potential to serve as a vector.

In addition to *C. oxystoma*, we molecularly sequenced other species of the *C. schultzei* complex. To the best of our knowledge, these are the first reported sequences, other than those of *C. oxystoma*, representing the *C. schultzei* complex. Sequences of ITS-1 and COI in this other member of the species complex, showed 87 and 90% identity, respectively, to the available GenBank sequences of *C. oxystoma* from Japan. Phylogenetic analyses of this molecularly identified species place it in a separate clade and supports a different genetic population within the *C. schultzei* complex. Distribution studies of this distinct genetic group of the *C. schultzei* complex showed that it is mostly found in semiarid regions, suggesting an Afrotropical origin. Additional investigation of this species' genetic composition may further differentiate it into separate genetic groups and give a clue as to its origin.

Species distribution in this study revealed that both genetic clusters of the *C. schultzei* complex were less common or even absent in comparison with *C. imicola* in the sampled settlements located in the southern parts of the country (Negev Desert). This suggests that arid environmental conditions might be less suitable for this species complex's breeding and survival. On the other hand, midges from the *C. schultzei* complex are prevalent in Africa [15] and have been found in the Sinai Desert [41], [42]. Thus, it is possible that the data in our study represent genetic populations that differ from those found in Africa, or that different trapping methods reveal different species assemblies.

The wide distribution of *C. oxystoma* described herein places this potential vector species as an important *Culicoides* species in Israel. The molecular findings for *C. oxystoma* in this study may serve in future studies of vectoring capacity and epidemiology of *Culicoides*-borne diseases and their prevention.

## Methods

### Ethics statement

No specific ethics permits were required for the described studies. The collection of midges were coordinated and permitted by the farm owners. Collections were not performed in national parks or other protected area of land, and did not involve endangered or protected species.

### Collection of adult *Culicoides* species

Midges were collected from 15 settlements located in different geographical and climatic regions throughout Israel during the summers of 2009–2010 (Table 1) using UV-light traps with a suction fan (John W. Hock Company, Gainesville, FL, USA) placed 1.5 to 2 m above the ground on cattle or horse farms. Insects were collected overnight into a plastic beaker containing absolute ethanol and kept at 4°C until analysis.

**Table 1:** Geographic trap locations used in this study.

Climatic region	Altitude (m)	Geographic coordinates	Trap location	No.
Mediterranean	185	33°01′16″N, 35°34′37″E	Ayelet Hashahar	1
Mediterranean	138	32°54′17″N, 35°32′59″E	Vered Hagalil	2
Semiarid	−185	32°35′15″N, 35°33′13″E	Neve Ur	3
Mediterranean	27	32°24′06″N, 34°55′46″E	Givat Haim	4
Mediterranean	22	32°22′49″N, 34°52′29″E	Kfar Vitkin	5
Mediterranean	29	32°11′56″N, 34°49′36″E	Rishpon	6
Mediterranean	52	32°02′32″N, 34°49′31″E	Ramat Gan	7
Arid	−215	32°00′00″N, 35°26′43″E	Gilgal	8
Mediterranean	82	31°57′23″N, 34°55′31″E	Ben Shemen	9
Mediterranean	77	31°55′20″N, 34°49′21″E	Netzer Sereni	10
Mediterranean	54	31°50′45″N, 34°48′34″E	Beit Elazari	11
Mediterranean	61	31°43′07″N, 34°43′45″E	Kfar Warburg	12
Arid/Semiarid	97	31°18′14″N, 34°31′09″E	Urim	13
Arid/Semiarid	117	31°16′50″N, 34°27′05″E	Ein HaBesor	14
Arid	120	29°53′46″N, 35°03′34″E	Yotvata	15

Two of the 15 settlements were used as collection sites for phylogenetic analysis. The first site, Neve-Ur, is located in the northern Jordan valley (south of the Sea of Galilee, in a semiarid region), and the second site, Beit-Elazari, is located in the center of the country (Mediterranean region). To study the geographical distribution of the molecularly defined species, we sampled midges from all settlements.

### Morphological identification

Initial identification of *Culicoides* species was based on wing-spot pattern and other morphological keys [8]. While *C. imicola* wings are pale with darker markings, *C. schultzei* complex wings are characterized by two pale spots. Wings, head and abdomen of individual midges were mounted in a mixture of Canada balsam and phenol on microscope slides for morphological identification [43], leaving thorax and legs for subsequent DNA extraction [29].

### Genomic DNA extraction and PCR amplification

Individual midges were washed with sterile double-distilled water and ground using a motorized hand homogenizer (pellet-pestle; Sigma, St. Louis, MO, USA) in a microcentrifuge tube containing 50 µL ice-cold lysis buffer as described by Frohlich et al. [44].

For PCR amplification of approximately 400 bp of the ITS-1 region of *Culicoides*, genus-specific primers PanCulF 5′-GTAGGTGAACCTGCGGAAGG-3′ and PanCulR 5′-TGCGGTCTTCATCGAACCCAT-3′ were synthesized (Metabion, Martinsried, Germany) and used according to the published cycling profile as previously described [22]. Amplification of the COI gene was carried out using primers C1-J-1718m 5′-GGAGGATTTGGAAATTGATTAGT-3′ and C1-N-2191m 5′-CAGGTAAAATTAAAATATAAACTTCTGG-3′ to obtain an approximately 600-bp product as described previously [30].

Based on sequences alignment of the ITS-1 gene obtained in this work, species-specific primers were designed to amplify an approximately 300 bp PCR products for each of the two different genetic groups. In order to identify *C. oxystoma* specimens, primers OXY-F 5′-GTGTCGTCTTGTCACGACA-3′ and PanCulR were used. Parallel identification of the other genetic cluster of the *C. schultzei* complex was utilized using the primer pair: PanCulF and Schultzei-R 5′-TAACACACACACTAGGTRTRT-3′. The cycling profile used for both species-specific reactions was an initial denaturation stage of 5 minutes at 94°C followed by 30 cycles of 1 minute at 94°C, 30 seconds 53°C, 30 seconds 72°C and a final elongation of 10 minutes at 72°C.

All amplifications were carried out in a total reaction volume was 25 µL using Gotaq colorless master mix (Promega, Madison, WI, USA), 2 µL DNA and 1 µL of 10 µM of each primer. Amplification was performed in a thermal cycler (Biometra, Goettingen, Germany). PCR products were stained with EZ-vision (Amresco, Solon, OH, USA), loaded onto a 1.5% agarose gel and visualized under UV light.

### Cloning and sequencing

PCR products of each of the genes (ITS-1 and COI) of 12 individual midges (10 of *C. schultzei* complex, 2 of *C. imicola*) were cloned into the pCR2.1 TOPO TA Cloning System (Invitrogen, Carlsbad, CA, USA) and transformed into HIT Competent *Escherichia coli* DH5α (RBC Bioscience, Taipei, Taiwan). Transformed colonies grown on LB (Lysogeny broth) agar containing ampicillin, X-gal (5-bromo-4-chloro-indolyl-galactopyranoside) and IPTG (Isopropyl β-d-1-thiogalactopyranoside) were selected according to color differentiation. Clone inserts were reamplified using the above-described PCR. Inserts containing plasmids were isolated with the DNA-spin™ kit (Intron Biotechnology, Kyungki-Do, Korea). DNA sequencing was carried out utilizing the BigDye Terminator cycle sequencing chemistry from Applied Biosystems (ABI, Carlsbad, CA, USA), the 3700 DNA analyzer, and the ABI data-collection and sequence-analysis software (Center for Genomic Technologies, The Hebrew University of Jerusalem). Sequences were analyzed using DNAMAN (Lynnon Corporation, Pointe-Claire, Canada) and deposited in GenBank.

### Phylogenetic analysis

A total of 30 sequences of *Culicoides* ITS-1 available in GenBank (chosen according to representative clades identified in [28]), were used for phylogenetic analysis, including an outgroup and the sequences from 12 individual midges obtained in this study. In addition, phylogenetic analysis of a total of 21 COI gene sequences including 8 GenBank published *C. oxystoma*, one *C. imicola*, and the 12 midges obtained in this study was performed.

Multiple alignments of the DNA fragments were generated using MUSCLE [45]. Phylogenetic trees obtained from the alignments were created using the maximum likelihood method based on the Tamura 3-parameter best fit model [46] with 1000 bootstraps. The trees with the highest log likelihood are shown. The percentage of trees in which the associated taxa clustered together is shown next to the branches. All analyses were conducted in MEGA 5.10 [47]. In addition, neighbor joining trees were built (Data not shown).

### Species distribution

A total of 217 midges morphologically identified as members of the *C. schultzei* complex were sampled from the traps located in 15 settlements throughout Israel with an average of 15 individuals from each settlement (range: 2–39). In one of the settlements (Yotvata), *C. schultzei* complex midges were absent and only *C. imicola* midges were collected. *Culicoides* were then classified according to ITS-1 band size on the agarose gel into two groups: *C. oxystoma* and *C. schultzei* complex. Species distribution was demonstrated on a topographic-climatic map of Israel generated using ArcGis™ 9.3. Climate regions are based on the Köppen classification [48].

## References

[1] Mellor PS, Boorman J, Baylis M (2000). *Culicoides* biting midges: their role as arbovirus vectors.. Annu Rev Entomol.

[2] Kedmi M, Van Straten M, Ezra E, Galon N, Klement E (2010). Assessment of the productivity effects associated with epizootic haemorrhagic disease in dairy herds.. J Dairy Sci.

[3] Velthuis AG, Saatkamp HW, Mourits MC, de Koeijer AA, Elbers AR (2010). Financial consequences of the Dutch bluetongue serotype 8 epidemics of 2006 and 2007.. Prev Vet Med.

[4] Mayo CE, Gardner IA, Mullens BA, Barker CM, Gerry AC (2011). Anthropogenic and meteorological factors influence vector abundance and prevalence of bluetongue virus infection of dairy cattle in California.. Vet Microbiol.

[5] Mellor PS (1990). The replication of bluetongue virus in *Culicoides* vectors.. Curr Top Microbiol Immunol.

[6] Mellor PS, Boorman J (1995). The transmission and geographical spread of African horse sickness and bluetongue viruses.. Ann Trop Med Parasitol.

[7] Fu H, Leake CJ, Mertens PPC, Mellor PS (1999). The barriers to bluetongue virus infection, dissemination and transmission in the vector, *Culicoides variipennis* (Diptera: Ceratopogonidae).. Arch Virol.

[8] Institute for Animal Health hosted website.. http://www.culicoides.net/.

[9] Mathieu B, Perrin A, Baldet T, Delecolle JC, Albina E (2007). Molecular identification of western European species of *Obsoletus* complex (Diptera: Ceratopogonidae) by internal transcribed spacer-1 rDNA multiplex polymerase chain reaction assay.. J Med Entomol.

[10] Nolan DV, Carpenter S, Barber J, Mellor PS, Dallas JF (2007). Rapid diagnostic PCR assays for members of the *Culicoides obsoletus* and *Culicoides pulicaris* species complexes, implicated vectors of bluetongue virus in Europe.. Vet Microbiol.

[11] Gomulski LM, Meiswinkel R, Delecolle JC, Goffredo M, Gasperi G (2005). Phylogenetic relationships of the subgenus Avaritia Fox, 1955 including *Culicoides obsoletus* (Diptera, Ceratopogonidae) in Italy based on internal transcribed spacer 2 ribosomal DNA sequences.. Sys Entomol.

[12] Braverman Y, Messadeq N, Lemble C, Kremer M (1996). Reevaluation of the taxonomic status of the *Culicoides* spp. (Diptera: Ceratopogonidae) from Israel and the eastern mediterranean and review of their potential medical and veterinary importance.. J Am Mosq Control Assoc.

[13] Braverman Y, Barzilay E, Frish K, Rubina M, Barber TL, Jochim MM (1985). Bluetongue virus isolated from pools of *Culicoides* spp. in Israel during the years 1981–1983.. Bluetongue and related orbiviruses.

[14] Mellor PS, Osborne R, Jennings DM (1984). Isolation of bluetongue and related viruses from *Culicoides* spp. in the Sudan.. J Hyg (London).

[15] Cornet M, Brunhes J (1994). Revision des especes de *Culicoides* apparentees a *C. schultzei* (Enderlin, 1908) dans la region afrotropicale (Diptera, Ceratopogonidae).. Bull Soc Entomol Fr.

[16] Boorman J (1989). *Culicoides* (Diptera: Ceratopogonidae) of the Arabian peninsula with notes on their medical and veterinary importance.. Fauna of Saudi Arabia.

[17] Braverman Y, Boorman J, Kremer M (1976). Faunistic list of *Culicoides* (Diptera, Ceratopogonidae) from Israel.. Cah ORSTOM Ser Entomol Med Parasitol.

[18] Callot J, Kremer M, Braverman Y (1969). Notes sur des *Culicoides* recoltes en Israel (Diptera: Ceratopogonidae).. Bull Soc Pathol Exot.

[19] Cornet M (1981). Revision of the *Culicoides* species related to *Culicoides schultzei* (Enderlein), in the Ethiopian region-preliminary note.. Isr J Entomol.

[20] Wirth WW, Dyce AL (1985). The current taxonomic status of the *Culicoides* vectors of bluetongue viruses.. Prog Clin Biol Res.

[21] Braverman Y, Linley JR, Marcus R, Frish K (1985). Seasonal survival and expectation of infective life of *Culicoides* spp. (Diptera: Ceratopogonidae) in Israel, with implications for bluetongue virus transmission and a comparison of the parous rate in *C. imicola* from Israel and Zimbabwe.. J Med Entomol.

[22] Cetre-Sossah C, Baldet T, Delecolle JC, Mathiew B, Perrin A (2004). Molecular detection of *Culicoides* spp. and *Culicoides imicola*, the principal vector of bluetongue (BT) and African horse sickness (AHS) in Africa and Europe.. Vet Res.

[23] Cetre-Sossah C, Mathieu B, Setier-Rio ML, Grillet C, Baldet T (2008). Development and evaluation of a real-time quantitative PCR assay for *Culicoides imicola*, one of the main vectors of bluetongue (BT) and African horse sickness (AHS) in Africa and Europe.. Res Vet Sci.

[24] Deblauwe I, De Witte JC, De Deken G, De Deken R, Madder M (2011). A new tool for the molecular identification of *Culicoides* species of the *Obsoletus* group: the glass slide microarray approach.. Med Vet Entomol.

[25] Gomulski LM, Meiswinkel R, Delécolle JC, Goffredo M, Gasperi G (2006). Phylogeny of the subgenus *Culicoides* and related species in Italy, inferred from internal transcribed spacer 2 ribosomal DNA sequences.. Med Vet Entomol.

[26] Mathieu B, Delécolle JC, Garros C, Balenghien T, Setier-Rio ML (2011). Simultaneous quantification of the relative abundance of species complex members: application to *Culicoides obsoletus* and *Culicoides scoticus* (Diptera: Ceratopogonidae), potential vectors of bluetongue virus.. Vet Parasitol.

[27] Monaco F, Benedetto L, Di Marcello V, Lelli R, Goffredo M (2010). Development and preliminary evaluation of a real-time polymerase chain reaction for the identification of *Culicoides obsoletus* sensu strictu, *C. scoticus* and *C. montanus* in the *Obsoletus* Complex in Italy.. Vet Ital.

[28] Perrin A, Cetre-Sossah C, Mathiew B, Baldet T, Delecolle JC (2006). Phylogenetic analysis of *Culicoides* species from France based on nuclear ITS1-rDNA sequences.. Med Vet Entomol.

[29] Stephan A, Clausen PH, Bauer B, Steuber S (2009). PCR identification of *Culicoides dewulfi* midges (Diptera: Ceratopogonidae), potential vectors of bluetongue in Germany.. Parasitol Res.

[30] Dallas JF, Cruickshank RH, Linton YM, Nolan DV, Patakakis M (2003). Phylogenetic status and matrilineal structure of the biting midge, *Culicoides imicola*, in Portugal, Rhodes and Israel.. Med Vet Entomol.

[31] Nolan DV, Dallas JF, Mordue (Luntz) AJ (2004). Molecular taxonomy and population structure of a *Culicoides* midge vector.. Vet Ital.

[32] Pagès N, Sarto I, Monteys V (2005). Differentiation of *Culicoides obsoletus* and *Culicoides scoticus* (Diptera: Ceratopogonidae) based on mitochondrial cytochrome oxidase subunit I.. J Med Entomol.

[33] Wenk CE, Kaufmann C, Schaffner F, Mathis A (2011). Molecular characterization of Swiss Ceratopogonidae (Diptera) and evaluation of real-time PCR assays for the identification of *Culicoides* biting midges.. Vet Parasitol.

[34] Kaufmann C, Ziegler D, Schaffner F, Carpenter S, Pfluger V (2011). Evaluation of matrix-assisted laser desorption/ionization time of flight mass spectrometry for characterization of *Culicoides nubeculosus* biting midges.. Med Vet Entomol.

[35] Yanase T, Kato T, Kubo T, Yoshida K, Ohashi S (2005). Isolation of bovine arboviruses from *Culicoides* biting midges (Diptera: Ceratopogonidae) in southern Japan: 1985–2002.. J Med Entomol.

[36] Matsumoto Y, Yanase T, Tsuda T, Noda H (2009). Characterization of internal transcribed spacer (ITS1-ITS2) region of ribosomal RNA gene from 25 species of *Culicoides* biting midges (Diptera: Ceratopogonidae) in Japan.. J Med Entomol.

[37] Matsumoto Y, Yanase T, Tsuda T, Noda H (2009). Species-specific mitochondrial gene rearrangements in biting midges and vector species identification.. Med Vet Entomol.

[38] Mellor PS, Jennings DM, Braverman Y, Boorman J (1981). Infection of Israeli *Culicoides* with African horse sickness, bluetongue and akabane viruses.. Acta Virol.

[39] Braverman Y, Rubina M, Frish K (1981). Pathogens of veterinary importance isolated from mosquitoes and biting midges in Israel.. Insect Sci Appl.

[40] Braverman Y, Messaddeq N, Kremer M (1991). *Culicoides* (Diptera: Ceratopogonidae) vector species in Israel trapped from burrows of rodents that might be potential reservoirs of bluetongue and akabane viruses.. Congreso Internacional de las Asociaciones Sudoccidental - Europeas de Parasitologia.

[41] Braverman Y, Frish K, Rubina M (1979). Preliminary results of blood sucking arthropods survey in the Sinai peninsula.. Isr J Vet Med (Refuah Veterinarit).

[42] Braverman Y, Delecolle JC, Frish K, Rubina M, Kremer M (1981). New records of *Culicoides* species (Diptera: Ceratopogonidae) from Golan heights, Israel and Sinai peninsula.. Isr J Entomol.

[43] Wirth WW, Marston N (1968). A method for mounting small insects on microscope slides in Canada balsam.. Ann Entomol Soc Am.

[44] Frohlich DR, Torres-Jerez I, Bedford ID, Markham PG, Brown JK (1999). A phylogeographical analysis of the *Bemisia tabaci* species complex based on mitochondrial DNA markers.. Mol Ecol.

[45] Edgar RC (2004). MUSCLE: multiple sequence alignment with high accuracy and high throughput.. Nucleic Acids Res.

[46] Tamura K (1992). Estimation of the number of nucleotide substitutions when there are strong transition-transversion and G+C-content biases.. Mol Biol Evol.

[47] Tamura K, Peterson D, Peterson N, Stecher G, Nei M (2011). MEGA5: Molecular evolutionary genetics analysis using maximum likelihood, evolutionary distance, and maximum parsimony methods.. Mol Biol Evol.

[48] Goldreich Y (2003). The climate of Israel: observation, research and application.

